# Genetic identification of *Trichinella* species found in wild carnivores from the territory of Kazakhstan

**DOI:** 10.3389/fvets.2023.1266561

**Published:** 2023-09-14

**Authors:** Rabiga Uakhit, Anne Mayer-Scholl, Chincher Shin, Ainura Smagulova, Lyudmila Lider, Sergey Leontyev, Vladimir Kiyan

**Affiliations:** ^1^Laboratory of Parasitology, Department of Veterinary Medicine, S. Seifullin Kazakh Agrotechnical University, Astana, Kazakhstan; ^2^Laboratory of Biodiversity and Genetic Resources, National Center for Biotechnology, Astana, Kazakhstan; ^3^Department of Biological Safety, German Federal Institute for Risk Assessment, Berlin, Germany

**Keywords:** trichinellosis, parasitic transmission, wild animals, parasitology, Kazakhstan

## Abstract

Trichinellosis, also called trichinosis, is a foodborne parasitic disease caused by eating raw or undercooked meat from animals infected with *Trichinella* spp. larvae and affects both animals and humans. Although on the territory of Kazakhstan, the species characteristics and prevalence of this helminth were studied back in the 90s, the data have not been updated since then. Given the above, our study was aimed at identifying *Trichinella* spp. using parasitological and molecular genetics methods. In our work, we studied 160 samples of muscle tissue of wild animals living in the natural zones of steppes and semi-deserts. Of the animals examined, 32 were positive for *Trichinella* spp., including 1 lynx (*Lynx lynx*), 17 wolves (*Canis lupus*), 11 foxes (*Vulpes vulpes*), 1 jackal (*Canis aureus*) and 2 corsac foxes (*Vulpes corsac*). Helminths were extracted using the digestion method. DNA was extracted using a Gene Jet commercial kit (Thermo Fisher Scientific, United Kingdom). For species identification a multiplex PCR, amplification of ESV, ITS1, and ITS2 genes regions was performed. After that, uniplex PCR was performed on the 5S rDNA and ITS1 genes region for sequencing analysis. The resulting sequences were subsequently used to construct a phylogenetic tree and the studied samples were identified as *Trichinella nativa* and *Trichinella britovi*. Thus, we can conclude that there is a circulation of two species of *Trichinella* in Kazakhstan, highlighting that constant control and monitoring of wild animals are necessary to prevent transmission and protect the health of people.

## 1. Introduction

Trichinellosis is a foodborne parasitic zoonotic disease caused by the consumption of raw or semi-raw meat of animals infected by larvae of this nematode. The disease is both a public health hazard and a food safety problem in many parts of the world (1, 2). *Trichinella* species have a wide range of hosts, but predators and omnivores are most affected (3, 4). After eating infected meat, larvae are released and penetrate the intestinal mucosa. They develop into adult worms within days. Males and females copulate and then females releasing newborn larvae after 1 week. These larvae travel *via* blood to the skeletal musculature where they either encapsulated or remain unencapsulated depending on the species (5).

To date, according to the literature, *Trichinella* species are divided into two clades: encapsulated (*T. spiralis, T. nativa, T. nelsoni, T. britovi, T. murrelli, T. patagoniensis, and T. chanchalensis*, *Trichinella* T6, T8, and T9) and non-encapsulated (*T. pseudospiralis, T. papuae*, and *T. zimbabwensis*) (6, 7).

In Kazakhstan, the problem of trichinellosis has not been studied well enough. *T. nativa, T. britovi*, and *T. pseudospiralis* are reported in wolves, jackal, foxes and cats living mainly in Southern Kazakhstan (5). One of the early studies on the spread of *Trichinella* is the work of Shaikenov. Their studies show pathogen infection of predators with *Trichinella* spp. in the deserts of Kazakhstan. As they note, the lowest infestation in predators was found in the sandy desert (3.7%), while the highest infestation of animals is noted in the mountainous (17.8–20.1%) and semi-desert (15.6–22.1%) zones (8).

Boev S.N. et al. described that the infestation of foxes (*Vulpes vulpes*) and corsac foxes (*Vulpes corsac*) in the steppe zone was 10.4 and 8.1%, respectively, and was inferior in terms of infestation of the same animals in the mountainous and semi-desert zones (9).

As was reported by Pozio (5), *T. nativa* and *T. britovi* circulate among foxes, wolves, jackals, martens, wild cats, lynxes, and wild boars in Kazakhstan. Also, *T. pseudospiralis* was recorded in corsac foxes, two rooks, and an eagle. Additionally, human trichinellosis has been documented following the consumption of meat from wild boars (10).

Wild carnivores such as wolves (*Canis lupus*), foxes (*Vulpes vulpes*), jackals (*Canis aureus*) and corsac foxes (*Vulpes corsac*) are native members of *Canidae* native to the Eurasia region (11). In Kazakhstan, the wild carnivore population is estimated to be around 14,000 animals (Unified Internet resource of the Ministry of Ecology, Geology and Natural Resources of the Republic of Kazakhstan, 2022) (12). These carnivores, at the top of the food chain, are excellent predators but also scavengers, and thus play an important role as a reservoir host of *Trichinella* species in the wild (13, 14).

The purpose of this article is to describe the occurrence of *Trichinella* spp. in wild animals of Kazakhstan and to study the species affiliation of the detected *Trichinella* larvae based on molecular analysis.

## 2. Materials and methods

### 2.1. *Trichinella* larvae sample collection

**Figure 1:**
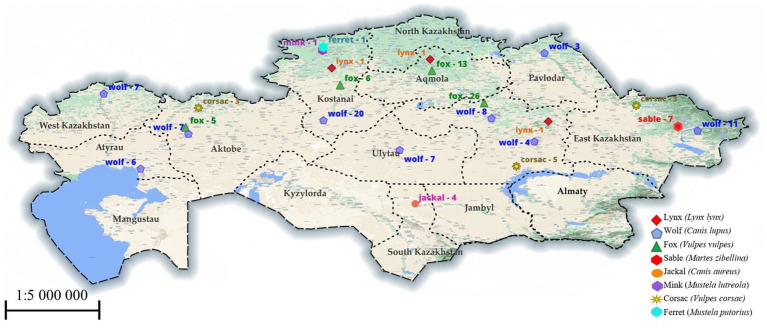
The region examined for *Trichinella* in animals. The numbers indicate the number of studied animals in a particular region.

Work with samples of muscle tissues of animals caught from nature was carried out in the parasitological laboratory of the Faculty of Veterinary Medicine and was approved by the Animal Ethics Committee of the S. Seifullin Kazakh Agrotechnical University (extract from protocol No. 1 dated July 24, 2019). We obtained permission from the Department of Natural Resources and Environmental Management to excavation of animals. The method that was used to remove them is through shooting. All procedures were in accordance with the World Medical Association Code of Ethics (Declaration of Helsinki) for animal experiments.^1^ The following animal species were studied: lynxes (*Lynx lynx*), wolves (*Canis lupus*), foxes (*Vulpes vulpes*), corsac foxes (*Vulpes corsac*), jackals (*Canis aureus*), mink (*Mustela lutreola*), ferret (*Mustela putorius*), sables (*Martes zibellina*), samples which were brought from 10 regions of Kazakhstan (Kostanay, Akmola, South Kazakhstan, Pavlodar, Karaganda, East Kazakhstan, West Kazakhstan, Aktobe, Atyrau and Ulytau regions), are shown in more detail in Figure 1. Animals were individually tested for the presence of *Trichinella* spp. larvae by the muscle compression method (15). Then, using artificial gastric juice, the muscle tissue (diaphragm and thigh muscle) was digested (16). The sediment was examined under a microscope, and *Trichinella* larvae were collected in test tubes with 70% ethanol. All morphological investigations were conducted using Micros Austria MCXI700.

### 2.2. DNA extraction

DNA was extracted from 310 larvae (as a pool of 10 larvae from each positive sample of an infected animal) using a GeneJet genomic DNA purification kit (Thermo Fisher, Cat.: K0701) with slight modifications. Briefly, proteinase K was added to the larvae and incubated at 48°C for 60 min, followed by all steps according to the manufacturer’s protocol.

### 2.3. Multiplex and uniplex PCR

A multiplex PCR based on the use of five primer pairs amplifying the internal transcribed spacers *ITS1* and *ITS2*, and the expansion segment V region (*ESV*) of the large subunit ribosomal DNA (17) was used. Uniplex PCR primers set (*5 s rDNA, ITS1*) for differentiating species by sequencing were utilized (18). Details of the *ESV*, *ITS1*, *ITS2* and 5 s rDNA fragments produced by the PCR amplification are shown in Table 1.

**Table 1:** Multiplex and uniplex PCR fragment size of the studied taxa of the genus *Trichinella*.

Primer	Locus set	Sequence (5′ → 3′)
I	ESV	F: GTTCCATGTGAACAGCAGT R: CGAAAACATACGACAACTGC
II	ITS1	F: GCTACATCCTTTTGATCTGTT R: AGACACAATATCAACCACAGTACA
III	ITS1	F: GCGGAAGGATCATTATCGTGTA R: TGGATTACAAAGAAAACCATCACT
IV	ITS2	F: GTGAGCGTAATAAAGGTGCAG R: TTCATCACACATCTTCCACTA
V	ITS2	F: CAATTGAAAACCGCTTAGCGTGTTT R: TGATCTGAGGTCGACATTTCC
VI	5S rDNA	F: GCGAATTCTTGGATCGGAGACGGCCTG R: GCTCTAGACGAGATGTCGTGCTTTCAACG

Reactions were performed in 15 μL 2^X^ GoTaq Hot Start MasterMix, 9 μL nuclease-free water, 1 μL total primers, and 2.5 μL extracted DNA. The uniplex PCR was performed using the same mix as above but with the primer set II and VI for the ITS1 and 5 s rDNA locus. The PCR cycles for multiplex PCR were performed as detailed in Table 2.

**Table 2:** Thermocycler parameters for 5S rDNA and multiplex primers gene amplification.

	Pre-denaturation		40 cycles		Final elongation
		Denaturation	Annealing	Elongation	
	Temp./Time				
Multiplex	95°C / 2 min	95°C / 10 s	55°C / 30 s	72°C / 30 s	72°C / 5 min
5 s rDNA	94°C / 1,5 min	94°C / 30 s	48°C / 1 min	72°C / 1 min	72°C / 10 min

### 2.4. Electrophoresis and sequencing

Agarose gels (1.5%) were prepared in 1× TAE solution with 8 ng/μL ethidium bromide (Sigma, E1510). Electrophoresis was performed using 10 μL PCR products with a DirectLoad 100 bp Low ladder ready-to-use (Sigma, D3687-1VL) for 50 min at 120 V. The PCR-amplified target gene fragment was purified using a QIAquick PCR Purification Kit (QIAGEN, Germany, Cat.: 28106), following the manufacturer’s protocols. Sequencing was performed according to the manual for Seq Studio Genetic Analyzer (Thermo Fisher Scientific Applied Biosystems). The resulting nucleotide sequences were visually checked by the Bio Capt program version 11.0. The nucleotide sequences of the studied species were compared with other sequences in the NCBI gene bank database by using the BLAST options. The nucleotide sequences of the studied species were deposited in NCBI GenBank database.

### 2.5. Bioinformatics analysis

Bioinformatic analysis of the obtained nucleotide sequences was carried out using computer software for statistical analysis of molecular evolution and construction of phylogenetic trees – MEGA 11. Multiple alignments were carried out using the CLUSTAL W program included in the MEGA software package. The evolutionary history and divergence between sequences was inferred by using the Maximum Likelihood method and Tamura-Nei model (19).

## 3. Results

**Figure 2:**
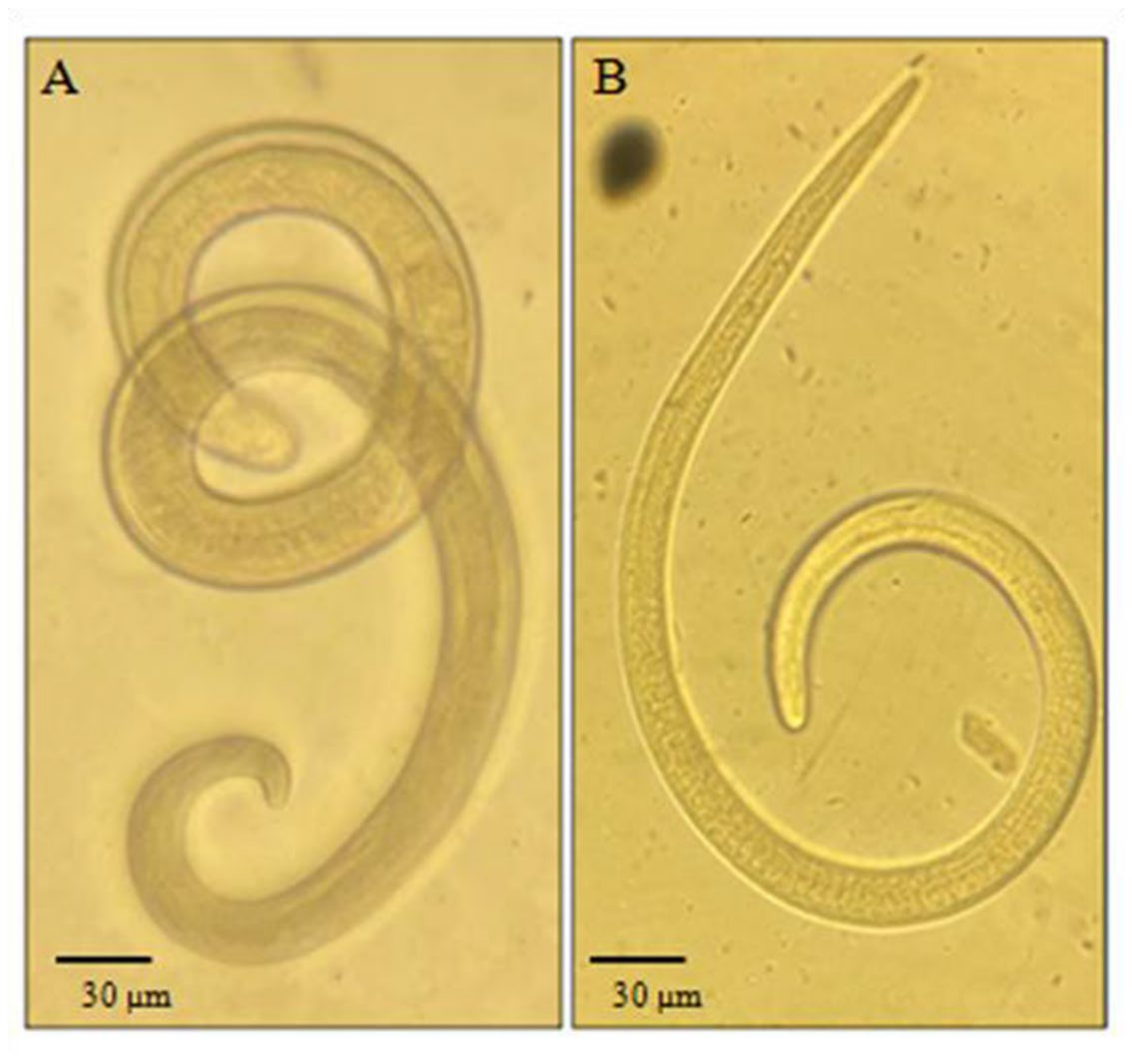
*Trichinella* larvae detected after digestion: **(A)** Specimen found in a lynx. **(B)** Specimen found in a fox. Magnification 10×.

Out of the 160 carnivores’ muscle tissue and diaphragm samples that were examined, 32 of them (20%, with a 95% confidence interval of 7.07–13.6) were found to be carriers of *Trichinella* larvae. Infection with *Trichinella* larvae was found in five out of the eight species that were examined. Specifically, 17 out of 83 wolves (20.5%), 11 out of 50 red foxes (22%), 2 out of 11 corsacs (18.2%), 1 out of 4 jackals (25%), and 1 out of 3 lynxes (33.3%) were infected, making a total of 32 samples. Examples of the detected helminths under the microscope can be seen in Figure 2. The specimens of ferret, sable, and mink that were examined were not infected with *Trichinella*. For more detailed information, please refer to Table 3.

**Table 3:** *Trichinella* infection, larval burden and species identification in wild animals in Kazakhstan.

Animal species	Examined (n)	Infected (n)	Infected (%)	grams of tissue examined for digestion (g)	95% CI (%)	LPG ± SD	*Trichinella* species
Lynx (*Lynx lynx*)	3	1	33.3	5	20 (−12–52)	8 ± 28.28	*T. nativa*
Wolf (*Canis lupus*)	83	17	20.5	5	12.59 (7.13–18.1)	12.5 ± 25.38	*T. nativa*
Fox (*Vulpes vulpes*)	50	11	22	5	9.2 (4.24–14.2)	9.5 ± 10.06	*T. nativa*
Corsac fox (*Vulpes corsac*)	11	2	18.2	5	3.8 (−1.1–8.7)	3.5 ± 8.3	*T. nativa*
Jackal (*Canis aureus*)	4	1	25	5	11.25 (−7.85–30.4)	9 ± 19.48	*T. britovi*
Mink (*Mustela lutreola*)	1	0	0	5	-	-	-
Ferret (*Mustela putorius*)	1	0	0	5	-	-	-
Sable (*Martes zibellina*)	7	0	0	5	-	-	-
Total	160	32	20		7.07–13.6	8.5 ± 21	

CI, confidence interval; LPG ± SD, larvae per gram of muscle tissue ± standard deviation.

**Figure 3:**
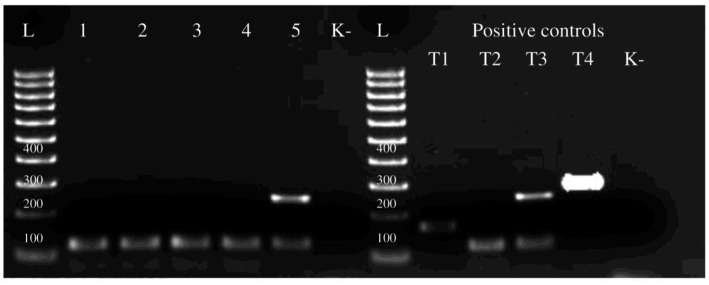
Electrophoretic profiles of *Trichinella* larva amplicons after multiplex PCR amplification: Lane L = 100 bp ladder, lane 1 – wolf, 2 – lynx, 3 – fox, 4 – corsac fox, 5 – jackal. Lane T1 – *T. spiralis*, T2 – *T. nativa*, T3 – *T. britovi*, T4 – *T. pseudospiralis* - positive control samples. K- – no-DNA control, ddH_2_O.

During the multiplex PCR, positive samples were found for *Trichinella nativa* in lynx, wolves, foxes, and corsac foxes (identified by amplification of a 127 bp fragment), while a jackal tested positive for *Trichinella britovi* (identified by amplification of two fragments at 127 and 253 bp) (as shown in Figure 3). To identify the specific species, additional PCR was conducted using primer 5 s rDNA and ITS1. The resulting amplicons were purified and sequenced for their nucleotide sequences.

**Figure 4:**
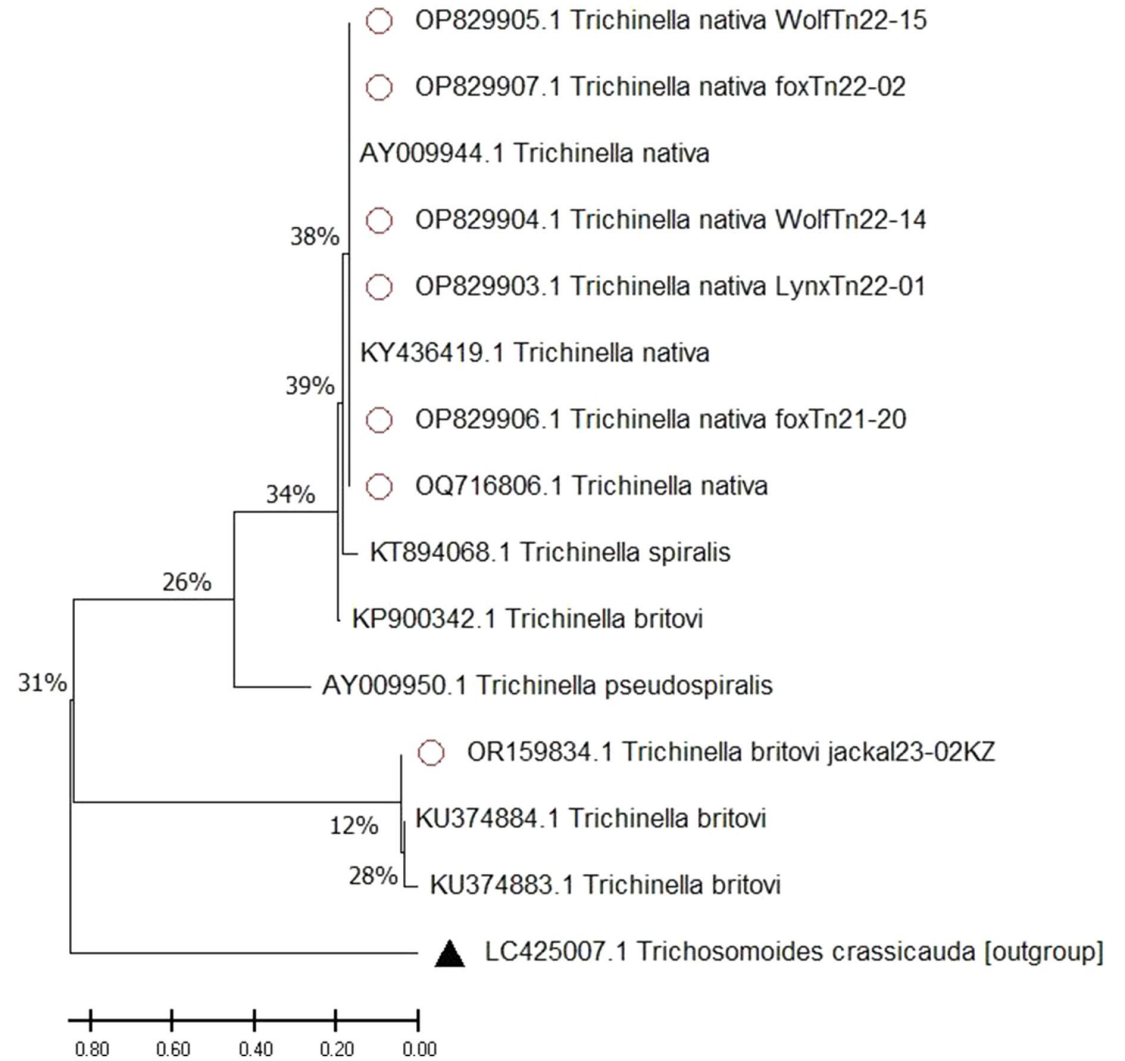
Maximum likelihood tree of *Trichinella* spp. A scale bar (divergence of 0.80) is shown. Sequences with red dots obtained in this study, outgroup marked with triangle, without any marks reference samples from GenBank.

The resulting nucleotide sequences confirmed that all *Trichinella* samples were *T. nativa* and *T. britovi*, and were deposited in the international GenBank database (OP829907, OP829905, OP829906, OP829903, OP829904, OQ716806, OR159834). The tree with the highest log likelihood (−4977.99) is presented (Figure 4), and the initial tree was obtained using the Maximum Parsimony method. The tree is to scale, with branch lengths measured in the number of substitutions per site. The proportion of sites where at least 1 unambiguous base is present in at least 1 sequence for each descendant clade is shown next to each internal node in the tree.

Our analysis involved 15 nucleotide sequences. The final dataset had a total of 1800 positions. The tree was rooted with an outgroup of *Trichosomoides crassicauda*. We also included sequences from *Trichinella* spp. species to show how closely related they are. We made sure to include *T. nativa* species from countries bordering Kazakhstan, such as China and Russia, where these species have been identified.

Obtained sequence data were used to calculate a pairwise fixation index (Fst), which helped us determine the level of genetic differentiation between *T. nativa* and *T. britovi* samples collected from carnivorous animals in Kazakhstan. Our analysis showed that the Fst values ranged from 0 to 1.084045 (as shown in Table 4), with 0 indicating complete identity among the samples.

**Table 4:** Pairwise fixation index (Fst values) between *T. nativa* and *T. britovi*.

	OP829905	OP829904	OP829907	AY009944	OP829906	OQ716806	OP829903	KY436419	KP900342	KT894068	AY009950	LC425007	OR159834	KU374884	KU374883
OP829905 *T. nativa* wolf															
OP829904 *T. nativa* wolf	0.00000														
OP829907 *T. nativa* fox	0.00000	0.00000													
AY009944 *T. nativa*	0.00000	0.00000	0.00000												
OP829906 *T. nativa* fox	0.00000	0.00000	0.00000	0.00000											
OQ716806 *T.nativa* corsac-fox	0.00000	0.00000	0.00000	0.00000	0.00000										
OP829903 *T.nativa* lynx	0.00628	0.00628	0.00628	0.00628	0.00628	0.00628									
KY436419 *T. nativa*	0.00628	0.00628	0.00628	0.00628	0.00628	0.00628	0.00000								
KP900342 *T. britovi*	0.01905	0.01905	0.01905	0.01905	0.01905	0.01905	0.02547	0.02547							
KT894068 *T. spiralis*	0.05872	0.05872	0.05872	0.05872	0.05872	0.05872	0.05871	0.05871	0.06592						
AY009950 *T. pseudospiralis*	0.37745	0.37745	0.37745	0.37745	0.37745	0.37745	0.37537	0.37537	0.36630	0.36492					
LC425007 *Trichosomoides crassicauda*	1.00884	1.00884	1.00884	1.00884	1.00884	1.00884	1.03736	1.03736	1.02709	1.039606	1.31151				
OR159834 *T.britovi* jackal	1.12977	1.12977	1.12977	1.12977	1.12977	1.12977	1.10229	1.10229	1.04405	1.123990	0.92394	1.07820			
KU374884 *T. britovi*	1.12977	1.12977	1.12977	1.12977	1.12977	1.12977	1.10229	1.10229	1.04405	1.123990	0.92394	1.07820	0.00000		
KU374883 *T.britovi*	1.12977	1.12977	1.12977	1.12977	1.12977	1.12977	1.10229	1.10229	1.04405	1.123990	0.92394	1.07820	0.00000	0.00000	

Sequences with red dots obtained in this study.

## 4. Discussion

From 2020 to 2023, we conducted studies on the occurrence and species identification of *Trichinella*, common in wild animals of Kazakhstan. During the study, the muscle tissue and diaphragm of 160 animals were studied. Infection of animals with *Trichinella* larvae was 20%, i.e., 32 tests were positive. The greatest detection of helminths was in areas located in the zone of steppes and semi-deserts.

To genetically identify the detected *Trichinella* larvae using sequencing, analysis was performed on the 5 s rDNA large ribosomal subunit and ITS1 Internal transcribed spacer (18, 20–22). After conducting research on wild mammals in Kazakhstan, we found that the species *T. nativa* is predominantly distributed throughout the territory. However, in the southern region of the country, we discovered the presence of the species *T. britovi*.

The process of identifying differences between species involves building a phylogenetic tree using the maximum likelihood method. *Trichosomoides crassicauda* (LC425007) was used as an outgroup to create a rooted tree. It is important to note that the studied sequence of *T. britovi* (OR159834) with reference sequences was divided into a separate clade, as the sequences were obtained by the ITS1 gene. The studied sequences of *T. nativa* wolves, foxes, lynxes and corsac foxes (OP829907, OP829905, OP829906, OP829903, OP829904, OQ716806) show deviations from the most recent common ancestor. The most recent common ancestor is the reference sample of *T. spiralis* (KТ894068). The studied *T. nativa* sequences (Fst values of 0.00652%) are grouped together with reference samples (AY009944, KY436419), forming one subclade, with a difference between the last common ancestor of 38%.

In general, the use of the maximum likelihood method to create a phylogenetic tree with *Trichosomoides crassicauda* as an outgroup and reference specimens of *T. spiralis, T. pseudospiralis, T. britovi*, and *T. nativa* provided insight into the unique evolutionary history of these species. The distinction of *T. nativa* from wolves, foxes, and lynx from the most recent common ancestor highlights the importance of considering evolutionary relationships when studying the genetic makeup of a species.

Through the analysis of the pairwise fixation index, it was discovered that there were minor variations between *T. nativa* species extracted from a variety of animals such as wolves, foxes, corsac foxes, and lynxes. Additionally, there were variations in nucleotide sequences between *T. nativa* collected from foxes (OP829906) and lynxes (OP829903). These findings offer significant insights into the genetic diversity of these species and could assist researchers in comprehending the ecological and evolutionary aspects that determine their distribution in different animal populations.

According to the map data in Figure 1, the samples we studied were obtained from four natural zones in Kazakhstan: forest-steppe and steppe in the northern areas, and semi-desert and desert in the western, central, and southern regions. We believe that weather and natural conditions do not affect the distribution of *Trichinella* species among wild carnivores in Kazakhstan. Although fewer animal specimens were studied in the southern part of the country, we found the *T. britovi* species in only 1 out of 4 jackals from the South Kazakhstan region. However, in all other samples of muscle tissue from wild carnivores in regions such as Karaganda, Ulytau, Kostanay, East Kazakhstan, and West Kazakhstan, we identified the *T. nativa* species.

As reported by Pozio (10), *T. nativa* and *T. britovi* are present among various wild animals such as red and corsac foxes, wolves, jackals, martens, wild cats, lynxes and wild boars (8, 9, 23) in Kazakhstan. In 1975, *T. pseudospiralis* was found in corsac foxes, two ravens and an eagle from the Chimkent region (25, 26). In 2016, 20 cases of human trichinosis were detected in the Kyzylorda region reported by Kenzhebaev. Out of the 20 patients, two were children under 14 years old, 14 lived in urban areas and 6 in rural areas. This group infection outbreak was of an epidemiological nature (24). The source of the infection was wild boar meat killed by an amateur hunter. There are likely both “wild” and “synanthropic” foci of trichinosis in south-west Kazakhstan. These foci initially formed in local reservoirs where the infection accumulated and grew in wild animals due to the “predator–prey” principle, ultimately leading to a group human infection with *Trichinella*. The sources of infection for animals and humans with *Trichinella* in Kazakhstan are diverse, and the pathogen continues to circulate due to the population of various field rodents, predators, and wild omnivores (boars) in the region (27).

In reference to the current situation regarding trichinosis in border countries with Kazakhstan, recent research conducted in mainland China has revealed that out of the 16 isolates obtained, 13 were identified as *T. spiralis*. These samples were collected from pigs all across the country. The remaining two isolates were obtained from dogs and one from a cat, and these were identified as *T. nativa*. These were collected in northeast China. It is worth noting that *Trichinella* has been found in 15 different animal species, including but not limited to pigs, dogs, cats, rats, cows, foxes, and bears, and these are distributed throughout China (28). Furthermore, a study conducted in Russia by Glazunov YV et al. found that *T. spiralis* was present in 58.8% of badgers (*Meles meles*), 35.3% of brown bears (Ursusarctos), and 5.9% of wild boars (Susscrofa) (29).

In Kyrgyzstan, there have been reports of the discovery of *T. nativa* in red foxes (23). However, there is currently no information available regarding the infection of humans or domestic animals with *Trichinella*. In Uzbekistan, *T. britovi* was found in jackals (23), and there have been reports of a significant outbreak of trichinosis caused by consuming pork from a wild boar (Kairov, 1965; Nadzhimiddinov et al., 1965). However, there is no information available regarding cases of infection in domestic animals (10).

Our research findings revealed that *Trichinella*, a causative agent of a parasitic disease, is widely distributed among wild mammals in all provinces of our country. This indicates that the natural biocenoses of Kazakhstan are at risk of invasion by *Trichinella*. Even with a relatively small sample size of animals under study, the results demonstrate the wide distribution of this parasite. As such, there is a real risk of human infection with *Trichinella* in the territory where the animals were caught. The main cause of human infections is often related to the illegal hunting of wild animals such as wild boar and badger, where the meat of poached mammals is not subjected to veterinary and sanitary examination. This study is of utmost importance, as data on *Trichinella* prevalence in wildlife is scarce in our region. By gaining more information, we can develop strategies to prevent and control the spread of *Trichinella* in our natural biocenoses.

## Data availability statement

The datasets presented in this study can be found in online repositories. The names of the repository/repositories and accession number(s) can be found at: https://www.ncbi.nlm.nih.gov/genbank/, OP829907, https://www.ncbi.nlm.nih.gov/genbank/, OP829905, https://www.ncbi.nlm.nih.gov/genbank/, OP829906, https://www.ncbi.nlm.nih.gov/genbank/, OP829903, https://www.ncbi.nlm.nih.gov/genbank/, OP829904.

## Ethics statement

The animal study was approved by the Animal Ethics Committee S.Seifullin Kazakh Agrotechnical Uneversity (extract from the protocol No. 1 from 24.07.2019). All of the procedures complied with the Code of Ethics of the World Medical Association (Declaration of Helsinki) for animal experiments (http://ec.europa.eu/environment/chemicals/lab_animals/legislation_en.htm). The study was conducted in accordance with the local legislation and institutional requirements.

## Author contributions

RU: Formal analysis, Methodology, Software, Validation, Visualization, Writing – original draft. AM-S: Conceptualization, Methodology, Supervision, Validation, Writing – original draft, Writing – review & editing. CS: Writing – original draft, Formal analysis, Software, Validation, Visualization. AS: Writing – original draft, Formal analysis, Methodology, Software, Validation, Visualization. LL: Conceptualization, Methodology, Supervision, Validation, Writing – original draft. SL: Writing – original draft, Formal analysis, Methodology, Resources, Validation, Visualization. VK: Methodology, Project administration, Resources, Supervision, Writing – original draft, Writing – review & editing, Conceptualization, Data curation, Funding acquisition, Investigation.

## Footnotes

^1^ http://ec.europa.eu/environment/chemicals/lab_animals/legal_en.htm

## References

[1] Crisóstomo-Jorquera V Landaeta-Aqueveque C. The genus *Trichinella* and its presence in wildlife worldwide: a review. Transbound Emerg Dis. (2022) 69:e1269–79. doi: 10.1111/tbed.14554.1-11, PMID: 35398980

[2] Ricchiuti L Petrini A Interisano M Ruberto A Salucci S Marino L. First report of *Trichinella* pseudospiralis in a wolf (*Canis lupus* italicus). Int J Parasitol Parasites Wildl. (2021) 15:195–8. doi: 10.1016/j.ijppaw.2021.05.002, PMID: 34136345 PMC8182262

[3] Gottstein B Pozio E Nöckler K. Epidemiology, diagnosis, treatment, and control of trichinellosis. Clin Microbiol Rev. (2009) 22:127–45. doi: 10.1128/CMR.00026-08, PMID: 19136437 PMC2620635

[4] Borhani M Fathi S Harandi MF Simsek S Ahmed H Wu X. *Trichinella* infections in animals and humans of Iran and Turkey. Front Med. (2023) 10:1088507. doi: 10.3389/fmed.2023.1088507, PMID: 36817781 PMC9932804

[5] Pozio E. The impact of globalization and climate change on *Trichinella* spp. epidemiology. Food Waterborne Parasitol. (2022) 27:e00154. doi: 10.1016/j.fawpar.2022.e00154, PMID: 35498552 PMC9052037

[6] Ribicich MM Fariña FA Aronowicz T Ercole ME Bessi C Winter M. Reprint of: a review on *Trichinella* infection in South America. Vet Parasitol. (2021) 297:109540. doi: 10.1016/j.vetpar.2021.109540, PMID: 34384644

[7] Zarlenga D Thompson P Pozio E. *Trichinella* species and genotypes. Res Vet Sci. (2020) 133:289–96. doi: 10.1016/j.rvsc.2020.08.012, PMID: 33199264

[8] Shaikenov B Tazieva ZC Hörning B. The etiology of sylvatic trichinellosis in Switzerland. Acta Trop. (1977) 34:327–30. German. PMID: 23655

[9] Boev SN Bondareva VI Sokolova IB Tazieva ZK. Trichinosis in Kazakstan. Wiad Parazytol. (1966) 12:519–25. Russian. PMID: 4224793

[10] Pozio E. World distribution of *Trichinella* spp. infections in animals and humans. Vet Parasitol. (2007) 149:3–21. doi: 10.1016/j.vetpar.2007.07.002, PMID: 17689195

[11] Feidas H Kouam MK Kantzoura V Theodoropoulos G. Global geographic distribution of *Trichinella* species and genotypes. Infect Genet Evol. (2014) 26:255–66. doi: 10.1016/j.meegid.2014.06.009, PMID: 24951834

[12] Bizhanova N Grachev Y Saparov K Grachev A. Distribution, abundance and some features of the ecology of large carnivores in Kazakhstan: analytical review. Eur J Ecol. (2017) 3:96–111. Russian. doi: 10.26577/EJE-2017-3-786

[13] Ch’ung-Sung F. Case of *Trichinella* infection caused by boar (*Sus scrofa*) meat in Kazakhstan. Med Parazitol. (1958) 27:447–9. Russian. PMID: 13589469

[14] Shaikenov BSH. Ecological border of distribution of *Trichinella* nativa Britov et Boev, 1972 and *T. nelsoni* Britov et Boev, 1972. Wiad Parazytol. (1992) 38:85–91. PMID: 1299068

[15] Barlow A Roy K Hawkins K Ankarah AA Rosenthal B. A review of testing and assurance methods for *Trichinella* surveillance programs. Food Waterborne Parasitol. (2021) 24:e00129. doi: 10.1016/j.fawpar.2021.e00129, PMID: 34458599 PMC8379475

[16] Li F Cui J Wang ZQ Jiang P. Sensitivity and optimization of artificial digestion in the inspection of meat for *Trichinella* spiralis. Foodborne Pathog Dis. (2010) 7:879–85. doi: 10.1089/fpd.2009.0445, PMID: 20524897

[17] Karadjian G Heckmann A Rosa G Pozio E Boireau P Vallée I. Molecular identification of *Trichinella* species by multiplex PCR: new insight for *Trichinella* murrelli. Parasite. (2017) 24:52. doi: 10.1051/parasite/2017053, PMID: 29219110 PMC5721686

[18] Bilska-Zając E Różycki M Chmurzyńska E Antolak E Próchniak M Grądziel-Krukowska K. First case of *Trichinella* nativa infection in wild boar in Central Europe – molecular characterization of the parasite. Parasitol Res. (2017) 116:1705–11. doi: 10.1007/s00436-017-5446-6, PMID: 28439686 PMC5429344

[19] Tamura K Stecher G Kumar S. MEGA11: molecular evolutionary genetics analysis version 11. Mol Biol Evol. (2021) 38:3022–7. doi: 10.1093/molbev/msab120, PMID: 33892491 PMC8233496

[20] Wang ZQ Li LZ Jiang P Liu LN Cui J. Molecular identification and phylogenetic analysis of *Trichinella* isolates from different provinces in mainland China. Parasitol Res. (2012) 110:753–7. doi: 10.1007/s00436-011-2549-3, PMID: 21773771

[21] Rombout YB Bosch S Van Der Giessen JW. Detection and identification of eight *Trichinella* genotypes by reverse line blot hybridization. J Clin Microbiol. (2001) 39:642–6. doi: 10.1128/JCM.39.2.642-646.2001, PMID: 11158122 PMC87791

[22] Li LZ Wang ZQ Jiang P Zhang X Ren HJ Cui J. Molecular identification of a *Trichinella* isolate from a naturally infected pig in Tibet. China Korean J Parasitol. (2011) 49:381–4. doi: 10.3347/kjp.2011.49.4.381, PMID: 22355205 PMC3279676

[23] Shaikenov BS Boev SN. Distribution of *Trichinella* species in the old world. Wiad Parazytol. (1983) 29:595–608.

[24] Kenzhebaev SA Ibragimov D Zhumalieva GO. Epizootology (epidemiology) of helminthozoonoses in the southwest of republic of Kazakstan. Russ J Parasitol. (2018) 12:27–32. Russian. doi: 10.31016/1998-8435-2018-12-2-27-32

[25] Pozio E Christensson D Steen M Marucci G La Rosa G Brojer C. *Trichinella* pseudospiralis foci in Sweden. Vet Parasitol. (2004) 125:335–42. doi: 10.1016/j.vetpar.2004.07.020, PMID: 15482889

[26] Sharma R Thompson P Elkin B Mulders R Branigan M Pongracz J. *Trichinella* pseudospiralis in a wolverine (*Gulo gulo*) from the Canadian north. Int J Parasitol Parasites Wildl. (2019) 9:274–80. doi: 10.1016/j.ijppaw.2019.06.005, PMID: 31289720 PMC6593184

[27] Shaikenov BS. Landscape features of the formation of trichinosis foci in Kazakhstan [Landshaftnye osobennosti formirovanie ochagov trihinelleza v Kazahstane] // Izvestiya NAN RK, (2007), Biological series No 3:57–65. Russian

[28] Bai X Hu X Liu X Tang B Liu M. Current research of Trichinellosis in China. Front Microbiol. (2017) 8:1472. doi: 10.3389/fmicb.2017.01472, PMID: 28824597 PMC5539376

[29] Glazunov YV Vinogradova YA. Epidemiology study of *Trichinella* spiralis infection in Tyumen region. Arch Razi Inst. (2023) 78:515–21. doi: 10.22092/ARI.2022.359951.2522, PMID: 37396729 PMC10314267

